# Antidepressants, sertraline and paroxetine, increase calcium influx and induce mitochondrial damage-mediated apoptosis of astrocytes

**DOI:** 10.18632/oncotarget.23302

**Published:** 2017-12-14

**Authors:** Chee-Kin Then, Kao-Hui Liu, Ming-Hsuan Liao, Kuo-Hsuan Chung, Jia-Yi Wang, Shing-Chuan Shen

**Affiliations:** ^1^ Graduate Institute of Medical Sciences, College of Medicine, Taipei Medical University, Taipei, Taiwan; ^2^ School of Medicine, College of Medicine, Taipei Medical University, Taipei, Taiwan; ^3^ Department of Dermatology, Taipei Medical University Shuang Ho Hospital, New Taipei City, Taiwan; ^4^ Department of Pharmacology, College of Medicine, National Taiwan University, Taipei, Taiwan; ^5^ Department of Psychiatry and Psychiatric Research Center, Taipei Medical University Hospital, Taipei, Taiwan; ^6^ Department of Psychiatry, School of Medicine, College of Medicine, Taipei Medical University, Taipei, Taiwan; ^7^ Department of Physiology, School of Medicine, College of Medicine, Taipei Medical University, Taipei, Taiwan; ^8^ Department of Dermatology, School of Medicine, College of Medicine, Taipei Medical University, Taipei, Taiwan; ^9^ International Master/Ph.D. Program in Medicine, College of Medicine, Taipei Medical University, Taipei, Taiwan

**Keywords:** antidepressants, calcium overload, mitochondrial damage, astrocyte apoptosis

## Abstract

The impacts of antidepressants on the pathogenesis of dementia remain unclear despite depression and dementia are closely related. Antidepressants have been reported may impair serotonin-regulated adaptive processes, increase neurological side-effects and cytotoxicity. An ‘astroglio-centric’ perspective of neurodegenerative diseases proposes astrocyte dysfunction is involved in the impairment of proper central nervous system functioning. Thus, defining whether antidepressants are harmful to astrocytes is an intriguing issue. We used an astrocyte cell line, primary cultured astrocytes and neuron cells, to identify the effects of 11 antidepressants which included selective serotonin reuptake inhibitors, a serotonin-norepinephrine reuptake inhibitor, tricyclic antidepressants, a tetracyclic antidepressant, a monoamine oxide inhibitor, and a serotonin antagonist and reuptake inhibitor. We found that treatment with 10 μM sertraline and 20 μM paroxetine significantly reduced cell viability. We further explored the underlying mechanisms and found induction of the [Ca^2+^]_i_ level in astrocytes. We also revealed that sertraline and paroxetine induced mitochondrial damage, ROS generation, and astrocyte apoptosis with elevation of cleaved-caspase 3 and cleaved-PARP levels. Ultimately, we validated these mechanisms in primary cultured astrocytes and neuron cells and obtained consistent results. These results suggest that sertraline and paroxetine cause astrocyte dysfunction, and this impairment may be involved in the pathogenesis of neurodegenerative diseases.

## INTRODUCTION

Astrocytes play vital roles in maintaining housekeeping functions of the nervous system (CNS), including homeostasis of the extracellular environment, neuronal metabolism, shaping of the brain microarchitecture, and regulation of neurotransmitters, such as glutamate [1]. Besides of neuronal impairment [2–4], astrocyte dysfunction was also contribute to the pathogenesis of neurodegenerative diseases through its effect on neuronal survival [5, 6]. For instance, accumulation of neuron-derived amyloid beta 1-42 in activated astrocytes indicates debris-clearing activity of astrocytes in response to Alzheimer’s disease (AD)-related degeneration of local dendrites and synapses [7]. In Parkinson’s disease (PD), astrocytes confer neuroprotection by providing the antioxidant, glutathione [8, 9] and clearing extracellular synuclein [10]; however, under pathological conditions, astrocytes release toxic molecules [11] and proinflammatory cytokines which promote degeneration of nigral dopaminergic neurons [12].

The prevalence of antidepressant use has gradually increased around the world, especially the adverse of selective serotonin reuptake inhibitors (SSRIs) and serotonin-norepinephrine reuptake inhibitors (SNRIs) as first-line treatment options in depression [13]. Antidepressants elevate the transmission of one or more of the monoamines: serotonin, nor-adrenaline, or dopamine [14]. They are classified according to their mechanism of action: tricyclic and tetracyclic antidepressants (TCAs and TeCAs), SSRIs, serotonin antagonist and reuptake inhibitors (SARIs), monoamine oxidase inhibitors (MAOIs), and SNRIs. Antidepressants have been proved to improve cognitive functions [15–17] but there is also a large-scale clinical trial which determined no cognitive improvements in antidepressant users [18]. In addition, Andrews et al. proposed that antidepressants disrupt the serotonin-regulated adaptive process [19, 20] and worsen neurological side-effects, such as dizziness [21], drowsiness, and sedation [22]. Previous studies demonstrated that antidepressants cause cell death of normal cells [23, 24]. Furthermore, a meta-analysis by Moraros et al. revealed that antidepressant medication was associated with a 2-fold increase in odds of several cognitive impairments [25]. We also obtained consistent results of antidepressant medication being associated with an increased risk of dementia in our previous study [26]. According to those studies and integrating the idea of an astroglio-centric perspective of neurodegenerative diseases [6], we proposed to determine the effects of antidepressant on astrocytes in this study.

Both *in vitro* and *in vivo* studies proposed that astrocyte apoptosis could be triggered by several pathways, such as Ca^2+^ overload [27], mitochondrial dysfunction [28], oxidative stress [29], nuclear factor-κB (NF-κB) activation [30], endoplasmic reticulum stress [31], and protease activation [32]. Regulation of calcium is critical for astrocytic signaling [33, 34], while intense elevation of intracellular Ca^2+^ ([Ca^2+^]_i_) may be a possible mechanism linking antidepressants and astrocyte apoptosis. Mounting evidence also suggested calcium deregulation would lead to astrocytic cell death [27, 35–37] via reactive oxygen species (ROS) generation through activation of calpain and xanthine oxidase [30]. Moreover, Liu et al. previously revealed the fluoxetine induced apoptosis of astrocyte-derived glioblastomas via AMPAR-mediated calcium overload [38].

In this study, we evaluated the impacts of antidepressants on astrocyte survival and the underlying mechanisms. After screening 11 different antidepressants, we found that sertraline and paroxetine induced astrocyte apoptosis. Astrocyte apoptosis was mediated by elevation of [Ca^2+^]_i_, dysfunction of mitochondria, and activation of caspase, and was accompanied by ROS generation. Our exploration of molecular mechanisms of antidepressant-triggered astrocyte apoptosis in this study revealed that antidepressant medication might be a potential risk factor for neurodegenerative diseases.

## RESULTS

### Sertraline and paroxetine reduce astrocyte viability

**Figure 1:**
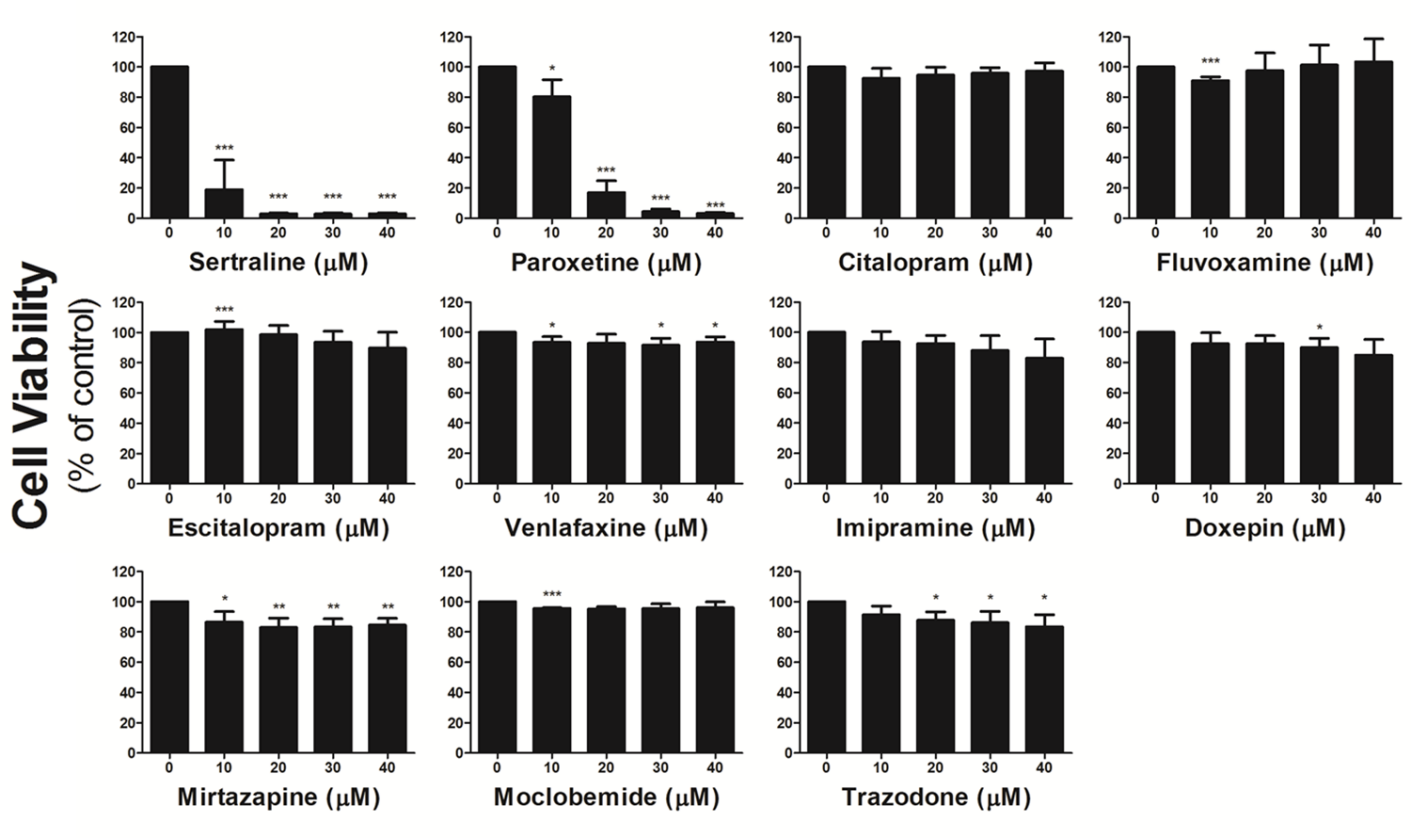
Sertraline and paroxetine reduce astrocyte viability Astrocyte viability was determined after treatment with indicated concentrations of antidepressants for 48 h by an MTT assay. Data were collected from three independent experiments and statistically analyzed by Student’s *t*-test, and results are shown as the mean ± SD. ^*^p<0.05, ^**^p<0.01, and ^***^p<0.001 compared to the control group.

We first investigated the effect of different antidepressants on the viability of an astrocyte cell line. As shown in Figure 1, we treated astrocytes with 0-40 μM of sertraline, paroxetine, citalopram, fluvoxamine, escitalopram, venlafaxine, imipramine, doxepin, mirtazapine, moclobemide, and trazodone for 48 h. The MTT results revealed that 10 μM sertraline or 20 μM paroxetine, two SSRIs, significantly reduced the cell viability of astrocytes. In contrast, we found no cytotoxicity toward astrocytes by the other antidepressants.

### Sertraline and paroxetine induce dose-dependent intracellular calcium elevation in astrocytes

**Figure 2:**
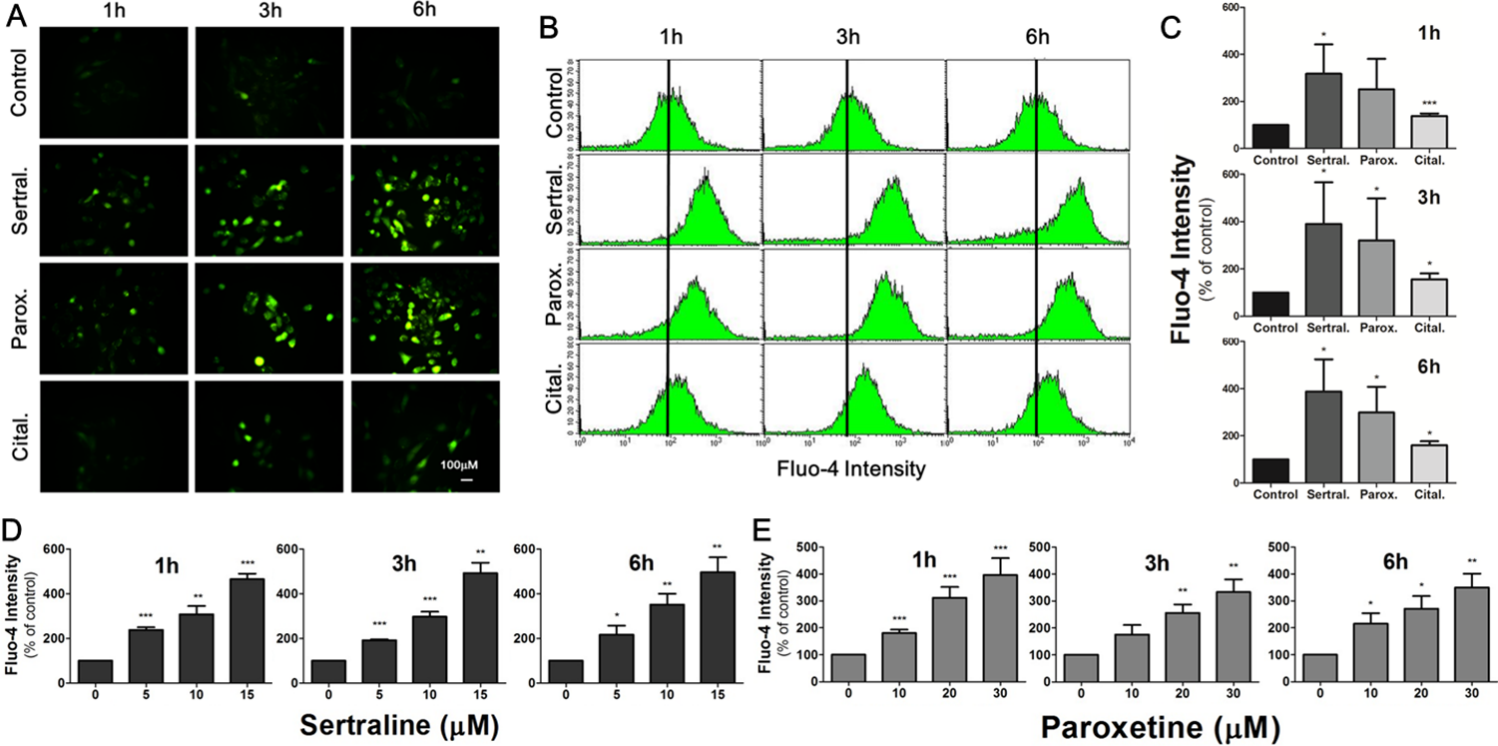
Sertraline and paroxetine induce dose-dependent intracellular calcium elevation in astrocytes **(A)** Fluorescence imaging of [Ca^2+^]_i_ using Fluo-4 was conducted after 1, 3, and 6 h of 10 μM sertraline, 20 μM paroxetine, and 20 μM citalopram treatment. **(B, C)** Quantitative data were collected by flow cytometry. Significant induction of fluorescence intensity was seen in cells exposed to sertraline and paroxetine compared to the control. **(D, E)** Sertraline and paroxetine triggered dose-dependent induction of intracellular calcium level. Data were collected from three independent experiments and statistically analyzed by Student’s *t*-test, and results are shown as the mean ± SD. ^*^p<0.05, ^***^p<0.001 compared to the control group.

We further studied the underlying mechanism of sertraline- and paroxetine-induced astrocyte cell death. It was reported that sertraline induces calcium levels in human prostate cancer cells. Thus, we used Fluo-4 as a calcium-sensitive probe to determine the influence of antidepressants on calcium flows. Treatment with sertraline and paroxetine increased the intracellular calcium concentration, and the calcium continued to rise in astrocytes at 3 and 6 h; however, only a slight increment in calcium levels was detected after citalopram treatment (Figure 2A). Calcium levels were quantified by flow cytometry (Figure 2B, 2C). Figure 2D and 2E further confirmed the dose-dependent effect of sertraline and paroxetine on the induction of intracellular calcium level.

### Sertraline and paroxetine induce mitochondrial membrane damage with depleted adenosine triphosphate (ATP) production and trigger ROS generation in astrocytes

**Figure 3:**
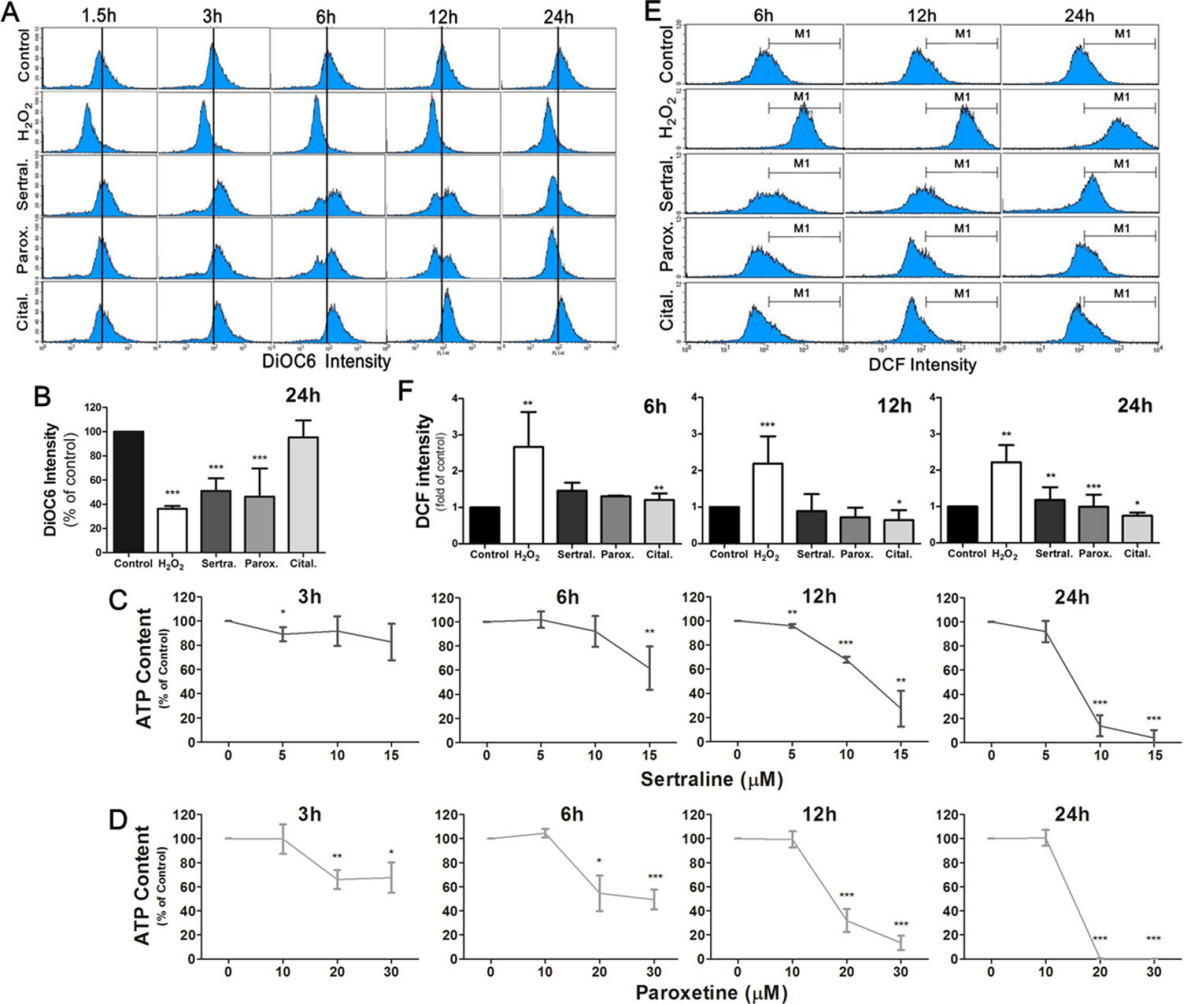
Sertraline and paroxetine induce mitochondrial membrane damage with depleted ATP production and trigger ROS generation in astrocytes **(A, B)** Astrocyte cells were treated with sertraline, paroxetine, and citalopram for 1.5, 3, 6, 12, and 24 h, and DiOC6 staining was used to examine the damage of mitochondrial membranes. The distribution of cells according to their mitochondrial membrane potential is shown in each panel of the figure, and means of DiOC6 intensity of different groups are compared in histograms. **(C, D)** ATP production was measured to monitor the mitochondrial function. **(E, F)** ROS generation was observed at 24 h after sertraline and paroxetine treatment. The DCF fluorescence intensity was measured by flow cytometric analysis, and the ratio of cells located at the M_1_ phase was quantified. H_2_O_2_ treatment is the positive control for ROS production. Data were collected from three independent experiments and statistically analyzed by Student’s *t*-test, and results are shown as the mean ± SD. ^*^p<0.05, ^***^p<0.001 compared to the control group.

Mitochondria are important organelles for storing calcium and play a key role in activating apoptosis. To examine the effects of sertraline and paroxetine on mitochondrial function, we used DiOC6 to determine the mitochondrial membrane potential (MMP) and applied mitochondrial functional assay to measure ATP content. Results revealed that induction of MMP at 3 h after treatment with sertraline and paroxetine, which indicated mitochondrial hyperpolarization. Figure 3A shows that treatment with sertraline and paroxetine decreased the MMP at 6 h and 12 h (third and fourth panels). Mitochondrial hypopolarization indicates that mitochondria have been damaged, occurred in most astrocytes of these two treated groups at 24 h (Figure 3A, 3B). Reduction of ATP production in sertraline- and paroxetine- treated astrocytes further confirmed that mitochondrial damage was occurred (Figure 3C, 3D). ROS-induced damage of mitochondria is one of the mechanisms involves in activating apoptosis. We found that both sertraline and paroxetine treatments significantly induced ROS production at 24 h (Figure 3E, 3F).

### Sertraline and paroxetine activate the intrinsic apoptotic pathway in astrocytes

**Figure 4:**
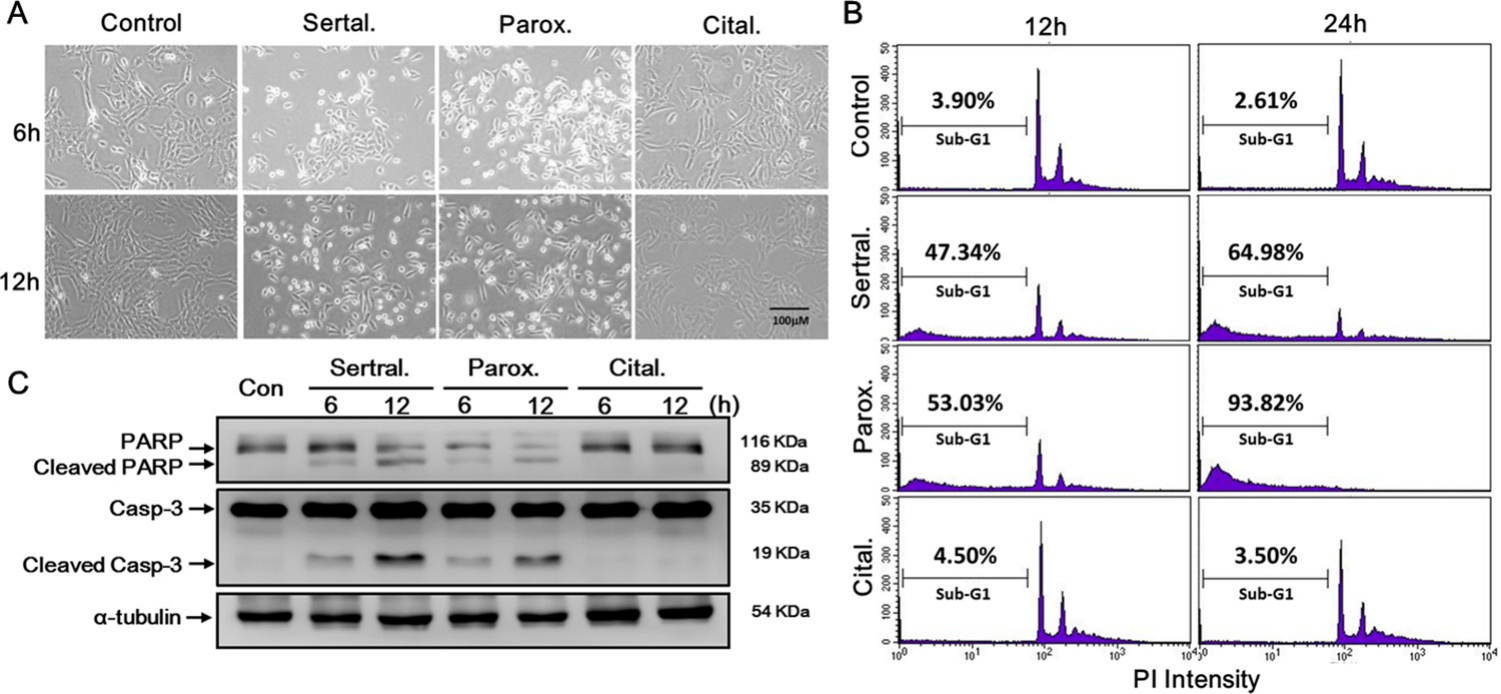
Sertraline and paroxetine activate the intrinsic apoptotic pathway in astrocytes Cell death was either **(A)** observed by microscopy or **(B)** quantified by a flow cytometric analysis. **(C)** Cleaved caspase-3 and cleaved poly(ADP-ribose) polymerase (PARP) proteins were determined by a Western blot analysis.

Images in Figure 4A show that the morphology of astrocytes had changed at 6 and 12 h in sertraline- and paroxetine-treated groups, but not in the citalopram-treated group. By a flow cytometric analysis, we also found that treatment with sertraline and paroxetine dramatically increased cell death, as indicated by the accumulation of sub-G_1_ population (Figure 4B). To define whether sertraline- and paroxetine-induced cell death was through apoptosis, we analyzed expressions of apoptosis-related proteins by a Western blot analysis. We found that treatment with sertraline and paroxetine, but not citalopram, induced cleavage of PARP and caspase-3 (Figure 4C). These data indicated that sertraline and paroxetine induced apoptotic signaling and cell death in astrocytes.

### Sertraline and paroxetine reduced cell viability in primary astrocytes, neurons, and their primary mixed culture

**Figure 5:**
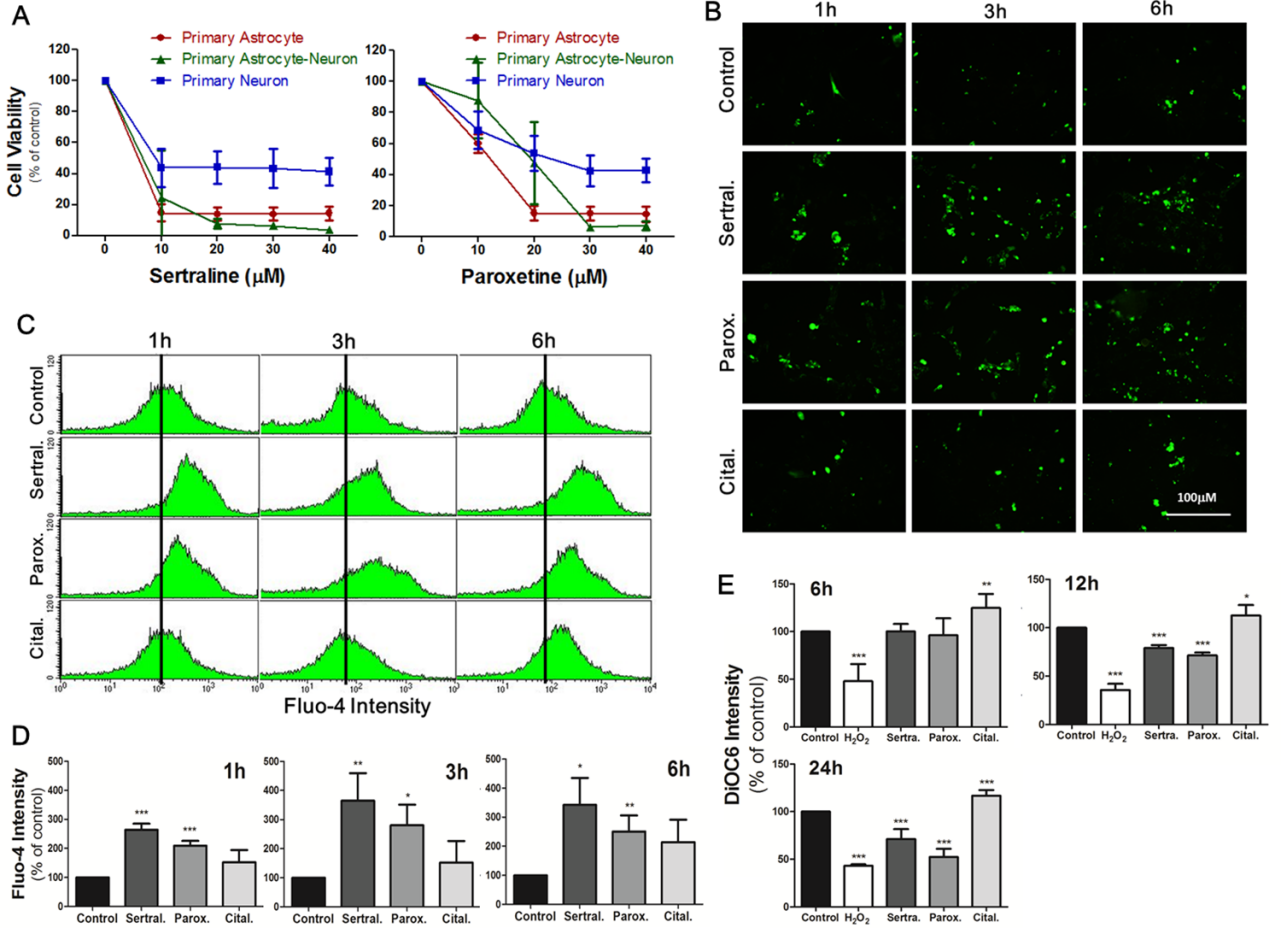
Sertraline and paroxetine reduced cell viability in primary astrocytes, neurons, and their primary mixed culture **(A)** Cell viability of different cells with indicated experimental conditions was detected by an MTT assay at 12 h after treatment. Data were collected from three independent experiments, and results are shown as the mean ± SD. **(B)** Fluorescence imaging of [Ca^2+^]_i_ of primary astrocytes using Fluo-4 was conducted after 1, 3, and 6 h of 10 μM sertraline, 20 μM paroxetine, and 20 μM citalopram treatment. **(C, D)** Quantitative data were collected by flow cytometry. **(E)** Mitochondrial damage of primary astrocytes was observed after 12 and 24 h of sertraline, paroxetine, and citalopram treatment. The DiOC6 fluorescence intensity was measured by a flow cytometric analysis. Data were collected from three independent experiments and statistically analyzed by Student’s *t*-test, and results are shown as the mean ± SD. ^*^p<0.05, ^***^p<0.001 compared to the control group.

To further confirm the aforementioned results, we used primary cultured astrocytes and neurons to establish a model to better mimic the nervous system. We further conducted experiments in primary astrocytes, primary neurons, and primary mixed astrocyte-neuron culture. The MTT assay showed that 10-40 μM sertraline had similar cytotoxic effects which significantly reduced primary astrocyte cell viability to <20%, while primary neuron cell viability remained around 50% (Figure 5A, left panel). Paroxetine showed consistent effects on primary cultured cells as did sertraline. Paroxetine at 20 μM significantly decreased the cell viability of primary astrocytes and primary mixed culture to around 20%, while the cell viability of primary neurons remained around 50% under the same conditions (Figure 5A, right panel). We further examined whether sertraline and paroxetine induced primary astrocyte cell death through the same mechanism in an astrocyte cell line. We observed that both sertraline and paroxetine induced intracellular calcium levels at 1, 3, and 6 h according to immunofluorescence staining (Figure 5B). We also found Ca^2+^ influx in primary cells in response to sertraline and paroxetine treatments by a flow cytometric analysis (Figure 5C, 5D). Sertraline and paroxetine also decreased the MMP in primary cells, which indicated consistent mitochondrial damage as in the astrocyte cell line (Figure 5E).

### Sertraline- and paroxetine-induced cell death was not associated with glutamate receptors, P2 receptor or TRPA1 receptor

**Figure 6:**
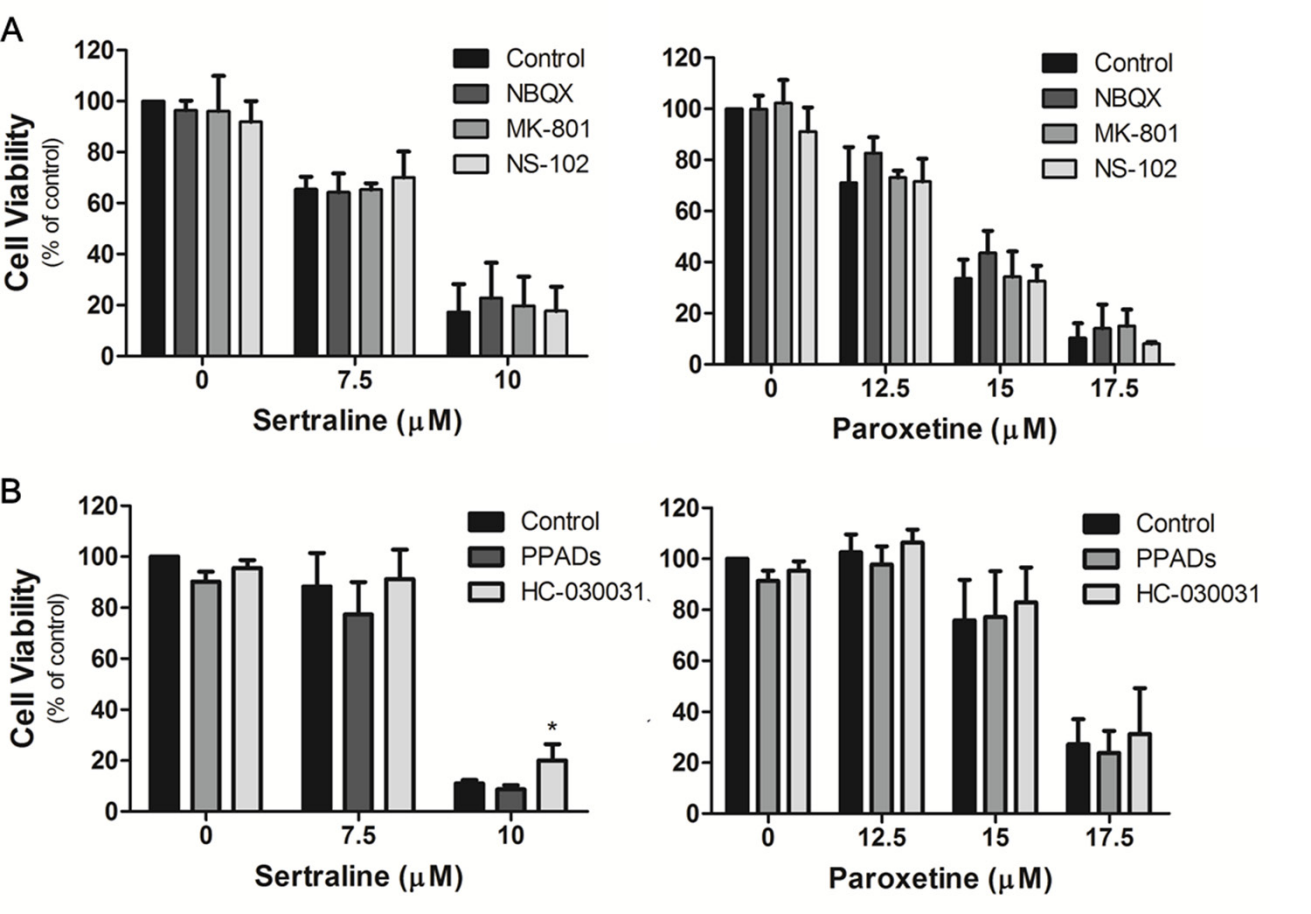
Sertraline- and paroxetine-induced cell death was not associated with glutamate receptors, P2 receptor or TRPA1 receptor Astrocytes were pretreated with **(A)** NBQX, MK-801, NS-102, **(B)** PPADs, and HC-030031, and then exposed to sertraline and paroxetine. Cell viability was analyzed by an MTT assay. Data were collected from three independent experiments and statistically analyzed by Student’s *t*-test, and results are shown as the mean ± SD. ^*^p<0.05, ^**^p<0.01, ^***^p<0.001 compared to the control group.

α-amino-3-hydroxy-5-methyl-4-isoxazolepropionic acid (AMPA) receptor (AMPAR) [39, 40], *N*-methyl-D-aspartate (NMDA) receptor [41], kainite receptor [42], P2 receptor [43, 44] and transient receptor potential ankyrin 1 (TRPA1) channel [45] are critical for astrocytic calcium influx. It was shown that fluoxetine induces calcium overload and causes apoptosis of glioma cells via activation of the AMPAR [38]. To examine whether sertraline and paroxetine also induce calcium overload via activation of the glutamate receptor, we pretreated astrocytes with several glutamate receptor inhibitors, including NBQX (AMPA/kainate receptor antagonist), NS-102 (kainate receptor antagonist), and MK-801 (NMDA receptor antagonist), followed by sertraline and paroxetine treatment. We found that none of these inhibitors rescued astrocytes from cell death (Figure 6A). We also pretreated astrocytes with the ATP-gated calcium channel inhibitors, PPADs (P2 receptor antagonist) and HC-030031 (TRPA1 channel antagonist), and found no protective effects of these two inhibitors on sertraline- and paroxetine-induced cell death (Figure 6B). These data suggest that sertraline- and paroxetine-induced cell death may occur through a glutamate receptor- and ATP-gated calcium channel-independent pathway.

## DISCUSSION

**Figure 7:**
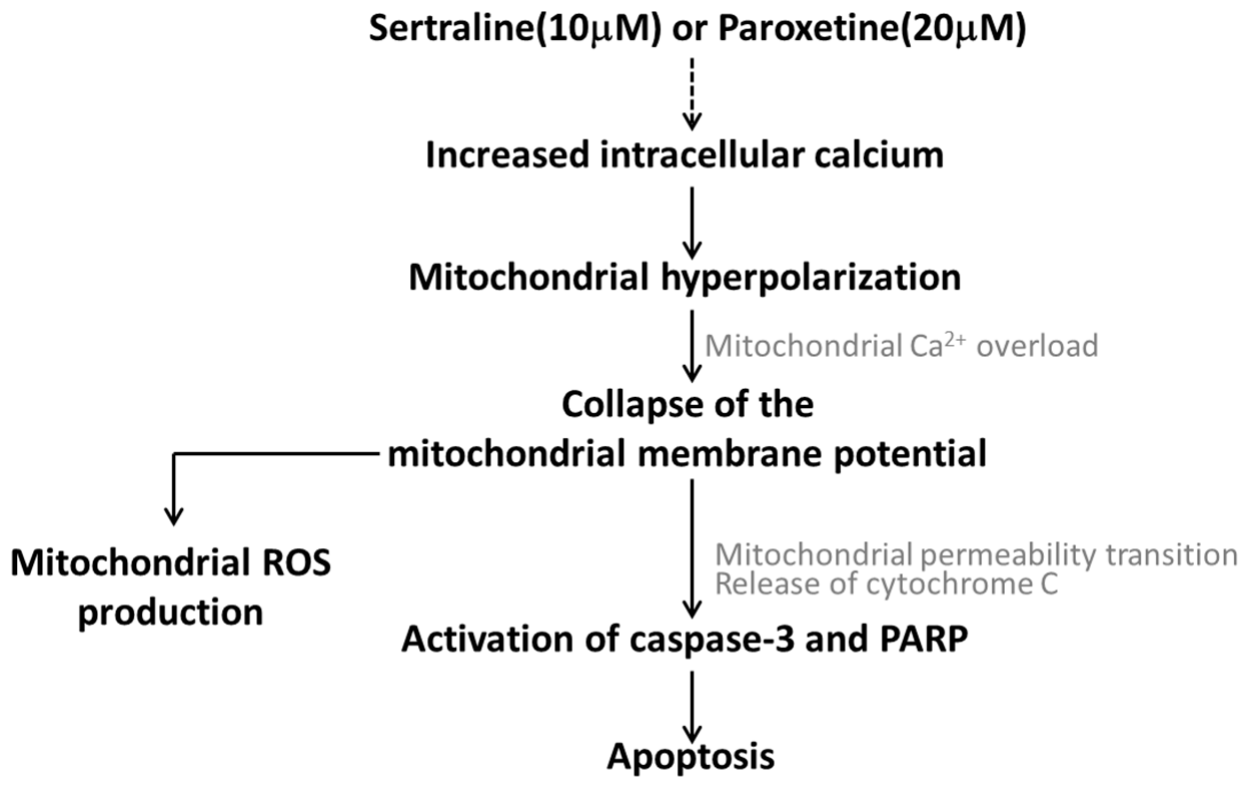
Working model related to sertraline- and paroxetine-induced astrocyte apoptosis Treatments with sertraline and paroxetine led to induction of intracellular calcium, mitochondrial hyperpolarization followed by mitochondrial damage, and reactive oxygen species (ROS) generation. Ultimately, caspase-3 and poly(ADP-ribose) polymerase (PARP) proteins were activated, and apoptosis occurred.

We found that sertraline and paroxetine, two SSRIs, reduced CTX TNA2 astrocyte viability but not the other nine commonly used antidepressants, which include three SSRIs, one SNRI, two TCAs, one TeCA, one MAOI, and one SARI. Sertraline and paroxetine initiated a complex response of astrocyte apoptosis. It is shown that calcium overload induced mitochondrial dysfunction, which eventually leads to the activation of an intrinsic apoptotic pathway (Figure 7). Consistent results of sertraline- and paroxetine-induced cell death and mitochondrial dysfunction were also observed in primary astrocytes.

At physiological levels, calcium wave is used for communication among neighboring astrocytes by gap-junctions and works together in the regulation of neuronal and non-neuronal functions of astrocytes, including synaptic neurotransmitter release, synaptic plasticity, and neuronal activity [34]. However, in a pathological state, an intracellular calcium rise is thought to play a primary role in initiating programmed cell death of neurons [46–48] and astrocytes [27, 49, 50]. Chiesa et al. suggested that perturbation of calcium homeostasis is sufficient to activate apoptosis in glial cells according to evidence of chromatin condensation, nucleus fragmentation, and genomic DNA laddering of astrocytes under a condition of calcium deprivation [49]. Astrocyte apoptosis was shown in several neurodegenerative diseases, including AD [51, 52]. Ultimately, astrocyte dysfunction renders them unable to execute housekeeping functions which further lead to neurodegenerative disorders.

Calcium influx is gated by several astroglial ionotropic receptors either directly (through receptor pores) or indirectly (by activating other Ca^2+^-permeable channels/transporters/exchangers), and they are the AMPAR [53, 54], NMDA receptor [54, 55], P2 receptors [54–56], TRPA1 channels [57], and etc. Lalo et al. demonstrated that AMPA and NMDA glutamate receptors [58] and P2×1/5 purinoceptor [59] are expressed by mouse cortical astrocytes, and their activation can evoke increased astrocytic [Ca^2+^]_i_ [55]. We observed the intracellular calcium rapidly rise at 1 h after treatment with sertraline and paroxetine. The dose-dependently induction of intracellular calcium levels by sertraline and paroxetine is also further support the idea that sertraline- and paroxetine-induced cell death of astrocyte due to calcium overload. However, the cell death of astrocytes could not be blocked by glutamate receptor inhibitors or ATP-gated ion channel inhibitors. Our results indicated that this calcium overload was not contributed to by activation of glutamate receptor or ATP-activated ion channel, since their blockage could not reverse antidepressant-induced astrocyte cell death. Therefore, we identified that intracellular Ca^2+^ elevation is invoked as a trigger for the apoptotic effects; however, the mechanism of sertraline- and paroxetine-induced calcium overload remains unclear. Astrocyte calcium signaling is critical for the release of gliotransmitters which act on neurons and vascular smooth muscles, while their dysregulation might be involved in several diseases including AD, HD, and epilepsy [60]. Therefore, whether sertraline and paroxetine disrupt astrocyte calcium signaling and further cause neurodegenerative diseases is an intriguing issue.

In addition, our results showed that sertraline and paroxetine caused mitochondrial hyperpolarization at 3 h. Two peaks respectively represent populations of hyperpolarization and hypopolarization of the MMP. This phenomenon indicated that astrocyte mitochondria were damaged after mitochondrial hyperpolarization, and this may possibly due to calcium overload. Previous studies showed several possible mechanisms of mitochondrial hyperpolarization, which included increased electron transport through mitochondrial respiratory chain (MRC) complexes [61], uncoupling of F0F1 ATP synthase [62], and cytosolic calcium rise [63]. Furthermore, we also showed the possibility of ROS formation may be a consequence of cell damage, not being the dominant pathway in inducing apoptosis.

Our previous article suggested that several antidepressant users were more likely to develop dementia, regardless their depression status [26]. Several studies showed consistent results with our observation and will be further discussed. A meta-analysis conducted by Moraros et al. revealed that antidepressant usage was associated with an increased odds of some types of cognitive impairment or dementia, and consistent results were revealed among two populations who were aged ≥65 (odds ratio (OR)=1.65) or <65 years (OR=3.25) [25]. In addition, Chen et al. demonstrated that sertraline activates the mitogen-activated protein kinase (MAPK) pathway and further triggers apoptosis in hepatic cells [23]. Mitochondrial dysfunction is also a possible mechanism of sertraline-associated hepatotoxicity [64]. Jason et al. indicated that sertraline and paroxetine significantly decreased cell viability and increased apoptosis of human osteoclasts and osteoblasts. Additionally, similar results indicated that citalopram was relatively safe to osteoclasts and osteoblasts, compared to the two other aforementioned SSRIs [24]. Consistent with our results proposing sertraline-induced calcium overload, Huang et al. demonstrated that sertraline induced a calcium rise in human PC3 prostate cancer cells, human OC2 oral cancer cells, and human MG63 osteosarcoma cells via its release from endoplasmic reticulum and multiple calcium influx pathways [65–67]. However, paroxetine evoked Ca^2+^-independent apoptosis via activation of p38 MAPK-associated caspase-3 and protein kinase C in human osteosarcoma cells [68] and renal tubular cells [69], respectively. Meanwhile, Taler et al. suggested that sertraline caused potent neurotrophic activity in human neuroblastoma cells and conferred a pro-cognitive effect by improving spatial learning memory [70]. The above study showed that lower concentration (below 10 μM) of sertraline and paroxetine enhanced cell viability of neuroblastoma cells [70]; however, in the present study, when the concentration of sertraline and paroxetine was elevated to more than 10 μM, the antidepressants significantly reduced cell viability. In addition, Peng et al. suggested that sertraline is neuroprotective in an HD mouse model from the aspect of improvement of motor performance and amelioration of brain atrophy. Steiner et al. demonstrated that paroxetine was neuroprotective against HIV Tat and gp120 and other mitochondrial toxins and enhanced proliferation of neural progenitor cells in a gp120 neurotoxicity transgenic model [71]. A recent article indicated the anti-inflammatory effect of paroxetine in the impairment of lipopolysaccharide-induced microglial activation [72].

Reis et al. showed that median serum concentrations of sertraline and paroxetine were 67 and 131 nM, respectively; however, no literature shows the information about antidepressant concentrations in the CNS [73]. These antidepressants might occur in low concentrations in the CNS; however, the development of astrocytic toxicity and further astrocyte-mediated neurotoxicity would be long-term processes and might result in brain degenerative changes. The prevalence of antidepressant use in Taiwan was up to 4.63% in 2009 [74]; hence, it is necessary to evaluate the safety of different classes of antidepressants. To the present, this is the first study to illustrate the possible mechanism of cytotoxicity of sertraline and paroxetine on astrocytes via calcium overload. This study is an extension of our previously published article on the potential risk of antidepressant medication [26]. Based on the fact that astrocyte dysfunction threatening the survival of neurons, we conclude that antidepressant-induced astrocyte apoptosis might be involved in the development of neurodegenerative diseases. More studies are warranted to further elucidate other possible effects and mechanisms of antidepressants in astrocyte death and neurodegeneration.

## MATERIALS AND METHODS

### Cell culture

The rat astrocyte CTX-TNA2 cell line was obtained from American Type Culture Collection and primary cortical neuron cells were obtained from Thermo Fisher Scientific. CTX-TNA2 cells were grown in Dulbecco’s modified Eagle’s medium (Invitrogen) supplemented with 10% heat-inactivated fetal bovine serum (Biological Industries). Primary astrocyte culture and astrocyte-neuron primary mixed cultures of postnatal day 1-3 Sprague-Dawley rats (BioLASCO Taiwan, Yilan, Taiwan) were prepared as previously described [75].

### Cell survival analysis

MTT assay was applied to assess cell viability. Briefly, 0.25 mg/mL MTT (Sigma) was added to cells at 37°C for 1 h, and the absorbance at 595 nm of MTT-formazan was detected spectrophotometrically using an ELISA reader (μQuant, Bio-Tek) after dissolution of the crystals in isopropanol. The percentage of cell viability was calculated by the formula: [Experimental group / Control group] x 100%.

### Cell cycle analysis by flow cytometry

To analyze the cell cycle of astrocyte cells, trypsin-EDTA was applied to dissociate cells from the culture dish, and cells were spun down at 8944 ×*g* for 5 min at 4°C. The supernatant was removed, and the cell pellet was suspended in 70% v/v ethanol at −20°C overnight. After the ethanol was removed by centrifugation, 0.5 mL of 0.5% Triton X-100 with RNase A (7 μg/ml) was used to suspend cell pellets, which were then incubated at 37°C for 30 min. Ultimately, 50 μg/ml propidium iodide (PI, Sigma) was added to the tube, and the fluorescent intensity at 637 nm was detected.

### Measurement of ROS generation by intact cells

Intracellular production of ROS by CTX-TNA2 cells was detected by oxidation of the probes DCFH-DA to DCF. DCFH-DA can readily enter cells due to its non-polar property. It is trapped within cells once it is hydrolyzed to the non-fluorescent polar derivative, DCFH. It turns into the highly fluorescent DCF if it undergoes oxidization. Before different treatments, cells were incubated in the dark for 1 h at 37°C with 50 μM DCFH-DA. Cells were harvested at 6, 12, and 24 h after treatment and were suspended in plain medium. CTX-TNA2 cells of each sample were analyzed, and the intracellular fluorescence was detected using a FACScan (Becton Dickinson, Sunnyvale, CA) flow cytometer with excitation at 488 nm and emission at 530 nm. The rise in peroxide levels was quantitated by measuring the percentage of cells at the M_1_ interval.

### Measurement of the mitochondrial membrane potential (MMP)

Cells were treated with the indicated concentration of sertraline and paroxetine for 1.5, 3, 6, 12, and 24 h. Before being harvested, cells were incubated with 40 nM DiOC6(3) (Sigma) for 30 min at 37°C. After that, cells were washed and suspended in phosphate-buffered saline (PBS). DiOC6(3) fluorescence intensities of FL-1 (530±15 nm) were measured with a flow cytometer CellQuest program (FACScan, Becton Dickinson).

### Cellular adenosine triphosphate (ATP) level measurement

ATP content, as a measurement of mitochondrial function, was detected and quantified by CellTiter-Glo Luminescent Cell Viability Assay (Promega Corporation, Madison, WI). The luminescent signals released from the assay were measured with a Spark™ 10M multimode microplate reader (Tecan Trading AG, Männedorf, Switzerland). The cellular ATP content was calculated by comparing the luminescence of the treated cells with that of the control.

### Intracellular calcium ([Ca^2+^]_i_) measurement

After treatment with antidepressants, cells were loaded with 5 μM Fluo-4, AM (Molecular Probes) for 30 min, and then real-time [Ca2^+^]_i_ images were immediately captured by fluorescence microscopy (Olympus IX81 microscope, NY, USA). To quantitate [Ca2^+^]_i_, cells were suspended in PBS, and the fluorescence intensities were measured with a flow cytometer (FACScan, Becton Dickinson) equipped with an excitation wavelength of 494 nm and an emission wavelength of 516 nm.

### Western blot analysis

Cell lysates were prepared by suspending cells in RIPA lysis buffer (50 mM Tris-HCl at pH 7.4, 1% Nonidet P-40, 150 mM sodium chloride, 1 mM sodium fluoride, 1 mM sodium orthovanadate, and 1 mM phenylmethylsulfonyl fluoride). An equal amount of protein was separated on 10% sodium dodecylsulfate-polyacrylamide gels and transferred to polyvinylidene difluoride membranes (Millipore). Membranes were probed with specific antibodies: anti-α-tubulin (Neomarker), anti-caspase-3 (Cell Signaling), anti-PARP (Imgenes) antibodies, and then probed with horseradish peroxidase (HRP)-labeled secondary antibodies. After enhanced chemiluminescent substrate (ECL) was added to the membrane, images were captured by the BioSpectrum Imaging System (AnAnalytik Jena Company, Ultra-Violet Products Ltd).

### Statistical analysis

Data are expressed as the mean ± standard deviation (SD), and each value in this study was repeated from three independent experiments. Statistical significance was determined using Student’s *t*-test. *p* values of <0.05, <0.01, and <0.001 were considered statistically significant differences.

## Abbreviations

AD: Alzheimer’s disease
HD: Huntington’s disease
PD: Parkinson’s disease
CNS: central nervous system (CNS)
SSRIs: selective serotonin reuptake inhibitors
SNRIs: serotonin-norepinephrine reuptake inhibitors
TCAs: tricyclic antidepressants
TeCA: tetracyclic antidepressant
SARIs: serotonin antagonist and reuptake inhibitors
MAOIs: monoamine oxidase inhibitors
ATP: adenosine triphosphate
IP3: inositol-1:4:5-trisphosphate
AMPA: α-amino-3-hydroxy-5-methyl-4-isoxazolepropionic acid
NMDA: N-methyl-D-aspartate
NF-κB: nuclear factor-κB
ROS: reactive oxygen species
PTP: permeability transition pore
MMP: mitochondrial membrane potential
PARP: poly(ADP-ribose) polymerase
MRC: mitochondrial respiratory chain
OR: odds ratio
MAPK: mitogen-activated protein kinase
PI: propidium iodide
PBS: phosphate-buffered saline
HRP: horseradish peroxidase
ECL: enhanced chemiluminescent substrate
SD: standard deviation
